# The National Medical Union

**Published:** 1914-02-14

**Authors:** Robert Carswell, E. Arthur Dorrell

**Affiliations:** Hon. Secretary; Hon. Secretary


### The National Medical Union.

A meeting of the Organisation Committee was held at the offices of the Union, 346 Strand, on February 7, Dr. Thomas Johnston, of Hove, in the chair.

It was reported that, so far, in only one or two districts has the classification of non-panel men been carried out with any degree of thoroughness. In these districts, however, the result has been encouraging and full of interest. Complete non-panel lists having been obtained, a systematic canvass has been carried out, the work being divided amongst energetic members of the local committees, who, armed with prospectuses and other information, have succeeded in enrolling a considerable proportion of their men. It has been found, as was to be expected, that there are a number of men who prefer to wait and see what is going to happen, and others who announce that they will join nothing, besides institution men, retired practitioners, locums, ship surgeons, and so on, though amongst the latter many were found to take a keen interest in the work that is being done.

The meeting decided to organise a similar canvass and classification of the whole profession, and arranged sub-committees for the purpose, which during the next month or two will be engaged in ensuring that every medical man who is not on the panel will at least have an opportunity of learning about the National Medical Union and of joining it.

A meeting of the sub-committee for this purpose in London has been called for Saturday, February 14, at 3.30 p.m., at the offices of the Union.

At a complimentary dinner to Dr. Addison on Friday last Mr. Lloyd George is reported to have stated that there were over 20,000 medical practitioners on the panels, "including doctors who were put on more than one panel."

A superficial comparison of the panel list for London with the lists for the surrounding counties shows immediately that a large number of names appear in two, or even more, county panels. The same applies with equal force to other large cities and to country Insurance areas. So serious a fallacy does this introduce into the figures given by the Chancellor that it would appear impossible to deduce from them any conclusion whatever as to the actual proportion of medical practitioners who have so far accepted service under the Insurance Acts.

When, in addition, it is recollected that at a meeting of the London Insurance Committee on January 22 Dr. H. H. Mills stated that there were about 1,000 doctors in London practising amongst the insured classes who had so far remained outside the Act, it will be very credible that a previous estimate of 14,000 for the total inclusive number of medical practitioners "on the panel" should be more nearly correct. Mr. Lloyd George's figures appear to be misleading to a degree.

Robert Carswell,
E. Arthur Dorrell, } Hon. Secretaries.

[We are requested to explain that the organisation referred to last week as the Islington Non-Panel Association should be more correctly described as the Islington and St. Pancras Non-Panel Association.]

